# Fish and macroinvertebrate assemblages reveal extensive degradation of the world's rivers

**DOI:** 10.1111/gcb.16439

**Published:** 2022-10-17

**Authors:** Maria João Feio, Robert M. Hughes, Sónia R. Q. Serra, Susan J. Nichols, Ben J. Kefford, Mark Lintermans, Wayne Robinson, Oghenekaro N. Odume, Marcos Callisto, Diego R. Macedo, Jon S. Harding, Adam G. Yates, Wendy Monk, Keigo Nakamura, Terutaka Mori, Masanao Sueyoshi, Norman Mercado‐Silva, Kai Chen, Min Jeong Baek, Yeon Jae Bae, Ram Devi Tachamo‐Shah, Deep Narayan Shah, Ian Campbell, Nabor Moya, Francis O. Arimoro, Unique N. Keke, Renato T. Martins, Carlos B. M. Alves, Paulo S. Pompeu, Subodh Sharma

**Affiliations:** ^1^ Department of Life Sciences, Marine and Environmental Sciences Centre ARNET, University of Coimbra Coimbra Portugal; ^2^ Amnis Opes Institute Corvallis Oregon USA; ^3^ Department of Fisheries, Wildlife, and Conservation Sciences Oregon State University Corvallis Oregon USA; ^4^ Centre for Applied Water Science Institute for Applied Ecology, University of Canberra Canberra Australia; ^5^ Charles Sturt University Albury New South Wales Australia; ^6^ Unilever Centre for Environmental Water Quality Institute for Water Research, Rhodes University Makhanda South Africa; ^7^ Departamento de Genética, Ecologia e Evolução Instituto de Ciências Biológicas, Universidade Federal de Minas Gerais Belo Horizonte Brazil; ^8^ Departamento de Geografia Universidade Federal de Minas Gerais Belo Horizonte Brazil; ^9^ School of Biological Sciences University of Canterbury Christchurch New Zealand; ^10^ Department of Biology University of Waterloo Waterloo Ontario Canada; ^11^ Environment and Climate Change Canada and Canadian Rivers Institute, Faculty of Forestry and Environmental Management University of New Brunswick Fredericton Canada; ^12^ Japan Riverfront Research Center Tokyo Japan; ^13^ Aqua Restoration Research Center Public Works Research Institute Kakamigahara Gifu Japan; ^14^ Centro de Investigación en Biodiversidad y Conservación Universidad Autónoma del Estado de Morelos Cuernavaca Morelos Mexico; ^15^ Department of Entomology Nanjing Agricultural University Nanjing People's Republic of China; ^16^ State Key Laboratory of Marine Resource Utilization in South China Sea Hainan University Haikou People's Republic of China; ^17^ National Institute of Biological Resources, Ministry of Environment Incheon Republic of Korea; ^18^ Division of Environmental Science and Ecological Engineering, College of Life Sciences Korea University Seoul Republic of Korea; ^19^ Department of Life Sciences, School of Science, Aquatic Ecology Centre Kathmandu University Dhulikhel Nepal; ^20^ Central Department of Environmental Science Tribhuvan University Kathmandu Nepal; ^21^ Rhithroecology Blackburn South Victoria Australia; ^22^ Instituto Experimental de Biologia Universidad Mayor Real y Pontificia de San Francisco Xavier de Chuquisaca Sucre Bolivia; ^23^ Applied Hydrobiology Unit, Department of Animal Biology Federal University of Technology Minna Nigeria; ^24^ Coordenação de Biodiversidade, Curso de pós‐graduação em Entomologia Instituto Nacional de Pesquisas da Amazônia Manaus Brazil; ^25^ Laboratório Nuvelhas, Projeto Manuelzão Universidade Federal de Minas Gerais Belo Horizonte Brazil; ^26^ Departamento de Ecologia e Conservação Universidade Federal de Lavras Lavras Brazil; ^27^ Aquatic Ecology Centre, School of Science Kathmandu University Dhulikhel Nepal

**Keywords:** anthropogenic degradation, biological assessment, climate, human development, human footprint, protected areas, streams

## Abstract

Rivers suffer from multiple stressors acting simultaneously on their biota, but the consequences are poorly quantified at the global scale. We evaluated the biological condition of rivers globally, including the largest proportion of countries from the Global South published to date. We gathered macroinvertebrate‐ and fish‐based assessments from 72,275 and 37,676 sites, respectively, from 64 study regions across six continents and 45 nations. Because assessments were based on differing methods, different systems were consolidated into a 3‐class system: Good, Impaired, or Severely Impaired, following common guidelines. The proportion of sites in each class by study area was calculated and each region was assigned a Köppen‐Geiger climate type, Human Footprint score (addressing landscape alterations), Human Development Index (HDI) score (addressing social welfare), % rivers with good ambient water quality, % protected freshwater key biodiversity areas; and % of forest area net change rate. We found that 50% of macroinvertebrate sites and 42% of fish sites were in Good condition, whereas 21% and 29% were Severely Impaired, respectively. The poorest biological conditions occurred in Arid and Equatorial climates and the best conditions occurred in Snow climates. Severely Impaired conditions were associated (Pearson correlation coefficient) with higher HDI scores, poorer physico‐chemical water quality, and lower proportions of protected freshwater areas. Good biological conditions were associated with good water quality and increased forested areas. It is essential to implement statutory bioassessment programs in Asian, African, and South American countries, and continue them in Oceania, Europe, and North America. There is a need to invest in assessments based on fish, as there is less information globally and fish were strong indicators of degradation. Our study highlights a need to increase the extent and number of protected river catchments, preserve and restore natural forested areas in the catchments, treat wastewater discharges, and improve river connectivity.

## Graphical abstract

The biological condition of rivers was assessed for 64 study regions of the world, based on macroinvertebrate and fish assemblages collected at 72,275 and 37,676 river sites, respectively. Globally, only 50% of macroinvertebrate sites and 42% of fish sites were in Good condition, whereas 21% and 29% were Severely Impaired. In some regions less than 25% of the river sites were in Good condition and more than 50% Severely impaired. In arid climates, Severely Impaired sites reach 51% for fish. Poorer biological conditions were associated with higher Human Development Index scores, poorer physico‐chemical water quality, and lower proportions of protected freshwater areas.

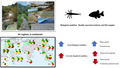

## 1 INTRODUCTION

Globally, rivers suffer from multiple anthropogenic pressures that typically act simultaneously (Best, 2019; Reid et al., 2019). These are driven by increased human populations and the pressures resulting from the increased consumption of goods and services (Díaz et al., 2019; IPBES, 2019; Reid et al., 2019). The result is excess of nutrients (Mekonnen & Hoekstra, 2017; USEPA, 2020), contaminants (Danner et al., 2019; Meng et al., 2020), altered flow regimes and connectivity (Belletti et al., 2020; Grill et al., 2019; Zarfl et al., 2015), impaired riparian vegetation and physical habitat structure (Aguiar et al., 2011; Kaufmann et al., 2022; Macfarlane et al., 2018), spread of invasive non‐native species (IPBES, 2019; Pereira & Ferreira, 2021; Seebens et al., 2017) and species overexploitation (Tickner et al., 2020).

Such stressors have caused greater losses of biodiversity in freshwaters than in terrestrial or marine systems (Collen et al., 2014; Darwall et al., 2018; Reid et al., 2019; WWF, 2020). They have degraded biological assemblage structure and composition (Chen & Olden, 2020; Dala‐Corte et al., 2020; Diaz et al., 2019), reduced functional diversity (Schmera et al., 2019), and altered ecosystem functioning (e.g., Pereira & Ferreira, 2021) leading to losses in ecosystem services. Such services include food and water provisioning, microclimate regulation (promoted by complex, layered riparian vegetation), improved water quality (promoted by aquatic and riparian vegetation and invertebrate and vertebrate filter feeders), and natural areas for recreation and relaxation (Díaz et al., 2019; Ranta et al., 2021; Riis et al., 2020).

Legislation and associated bioassessment programs have been developed for rivers across several continents and nations (Feio, Hughes, et al., 2021). The bioassessment is a critical instrument for evaluating status and trends in riverine health, conducting biological risk assessments, defining targets, elaborating cost‐effective restoration/rehabilitation measures, and evaluating the results of environmental policy and management actions. However, few nations have statutory bioassessment programs (Buss et al., 2015; Eriksen et al., 2021; Feio, Hughes, et al., 2021). Furthermore, the biological condition of rivers in the Global South (GS, sensu the United Nations' Finance Center for South–South Cooperation—excludes Australia and New Zealand and includes mostly low‐ to middle‐income countries of Africa, Asia, and South and Central America) is particularly understudied and most studies come from Brazil, Argentina, South Africa and China (Eriksen et al., 2021; Feio, Hughes, et al., 2021; Obubu et al., 2021). In many Asian, African, and Latin American countries riverine biological monitoring is lacking in terms of spatial and temporal coverage (Buss et al., 2015; Eriksen et al., 2021; Feio, Hughes, et al., 2021; Gallardo et al., 2018). These shortcomings have hindered rigorous global assessments of riverine biological conditions.

At the same time, there are indications that emerging threats to riverine biological conditions will be concentrated in the GS putting highly biodiverse regions at risk. For example, future hydropower development is concentrated in South America, South and Southeast Asia, and Africa (Couto et al., 2021; King & Brown, 2021; Zarfl et al., 2015). Dams and reservoirs alter river morphology, flow regimes, sediment transport, and riparian vegetation, thereby changing riverine habitats, creating barriers to fish migration, and displacing macroinvertebrates and other aquatic biota (Rivaes et al., 2022). In tropical areas, forests are severely threatened by deforestation for agricultural expansion (Hoang & Kanemoto, 2021). Forest removal decreases inputs of coarse organic matter that constitutes the basis of river food webs, decreases dry‐season flows and shade and increases water temperature. Increased loadings of fine sediments and nutrients occur from runoff over exposed agricultural soils. Such changes promote eutrophication, siltation, and water deoxygenation, which lead to the disappearance of sensitive taxa (Feld et al., 2018; Mainstone & Parr, 2002; Peña‐Arancibia et al., 2019). Also, 52% of the total phosphorus load to freshwaters occurs in Asia (30% from China), followed by Europe (19%) and Latin America and the Caribbean (13%) (Mekonnen & Hoekstra, 2017). China's Belt and Road Initiative (BRI), World Bank infrastructure funding (Perkins, 2005), increased and poorly regulated metal and fossil fuel mining (Hughes et al., 2016), and human overpopulation in the GS have the potential to degrade rivers in all the above ways (Gebremedhin et al., 2018; Sanon et al., 2020; Teo et al., 2019). Therefore, it is essential to raise the representativeness of rivers from the GS in global bioassessment studies.

Although some nations have implemented effective river rehabilitation programs, most others have done little to stem river degradation (Feio, Hughes, et al., 2021). In fact, important drivers of the freshwater biodiversity crisis are the disregard of the impacts of poor water management and land use on rivers by citizens and decision‐ and policy‐makers (Darwall et al., 2018; Pelicice & Castello, 2021). Thus, understanding the global patterns of riverine biological quality is essential for establishing global priorities for sustaining and restoring rivers and their ecosystem services. This is aligned with the United Nations sustainable development goals (SDG) of Agenda 2030, which aim to protect and enhance the quality of terrestrial and freshwater ecosystems, reverse the loss of aquatic biodiversity (SDG15), and reduce water pollution (SDG6).

The most‐used biological assemblages for assessing lotic ecosystem quality are benthic macroinvertebrates and fish (Davies et al., 2010; Eriksen et al., 2021; Feio, Hughes, et al., 2021; Ruaro et al., 2020). For example, the European Union Water Framework Directive (WFD, EC 2000) considers both as compulsory biological elements in the evaluation of rivers’ ecological quality. And they are also used in the Environmental and Monitoring Assessment Program of the United States Environmental Protection Agency and by the Sustainable Rivers Audit of the Murray–Darlin Basin in Australia (Davies et al., 2010). Both assemblages vary in their sensitivity and scale of response (temporal and spatial) to different stressors and complement each other because of their different characteristics (Herlihy et al., 2020; Morse et al., 2007). As bioindicators, macroinvertebrates have the advantages of ease of collection and are present in almost all aquatic habitats; their limited mobility makes them continuously subject to local conditions; species with life cycles long enough to reflect chronic effects of pollution, but short enough to respond to acute changes in the condition of the system (e.g., an effluent discharge); and there is a range of taxa with varying sensitivities to different types of anthropogenic disturbance (Bonada et al., 2006; Serra et al., 2019). Yet, their identification is time‐consuming and requires high taxonomic expertise. Fish are indicators of longer‐term alterations (years), as their larger movement capabilities and longer lifespans mean they integrate impacts over broader spatial and temporal scales. They are sensitive to water pollution and hydromorphological alterations; the environmental requirement of many fish species are better known than macroinvertebrates; they are socioeconomically important; and are relatively easy to collect and identify to species level and can be sorted in the field (Almeida et al., 2019; Barbour et al., 1999; Herlihy et al., 2020).

Here we analyzed the biological quality of the world's rivers and streams based on existing macroinvertebrate and fish bioassessment data from 64 study regions located in 45 nations and six continents/large regions (North and South America, Asia, Africa, Europe, and Oceania). In addition, we determined the correlations among anthropogenic pressures in the studied regions and the riverine biological quality assessment. We expected that regions with stronger and longer histories of modifications (excluding First Nations people), such as the European countries or other developed countries, would have more sites in poor biological condition, resulting from a greater period of simplification of aquatic communities, reduced abundances of sensitive taxa, and more invasive non‐native species (Chen & Olden, 2020; Harding et al., 1998; Herlihy et al., 2020; Rinne et al., 2005). The Human Footprint (WCS & CIESIM, 2005), and the Human Development Index (HDI) are well‐established indices that can be used for testing this hypothesis. In addition, the indicators of the implementation of Sustainable Development Goal 6 (SDG6), which focused on the sustainable management of water resources, wastewater and ecosystems, are expected to be correlated with the ecological quality of rivers. Yet, as far as we are aware, this has not been tested before. Finally, we also predicted that measures to protect rivers would be reflected in improvements in their biological condition (Haase et al., 2013; Krueger et al., 2019). Understanding the global biological condition of rivers and the relationships between their quality and the anthropogenic pressures on them should facilitate prioritization of river conservation efforts.

## 2 MATERIALS AND METHODS

### 2.1 Study regions

We gathered biological classifications for rivers and streams located in 64 regions across 45 nations (Table 1). In some regions the data covered entire or most of the countries (27 European nations, Japan, USA, New Zealand, South Africa, and South Korea). In other cases, data covered large hydrographic regions or major basins (Australia, Brazil, Cambodia, Canada, China, Laos, Mexico, Nepal, Nigeria, Thailand, and Vietnam). Predictably, data were scarcer from South Asia, Central and South America, and Africa.

**TABLE 1:** Distribution of sites by continent and study region (with respective codes) and the percent of sites in Good, Impaired (Imp) or Severely Impaired (SImp) condition based on benthic macroinvertebrate (INV) and fish (FISH) assemblages

Study regions		Number of sites		INV %			FISH %		
		INV	FISH	Good	Imp	SImp	Good	Imp	SImp
AF	South Africa (SA)	1426	NA	30	20	50	—	—	—
AF	Southern and North‐Central Nigeria (NI)	69	NA	30	42	28	—	—	—
AS	South Korea (SK)	1154	1156	52	31	17	39	45	16
AS	Upper Mekong—China (UL‐M)	176	NA	52	24	23	—	—	—
AS	Yangtze basin—China (YA)	483	NA	35	21	45	—	—	—
AS	Zhejiang coastal—China (ZC)	484	NA	46	23	31	—	—	—
AS	Zhu basin—China (ZH)	262	NA	37	15	48	—	—	—
AS	Japan (JA)	563	547	35	30	35	40	26	34
AS	Lower Mekong—Laos, Thailand, Vietnam, Cambodia (LM)	41	NA	66	32	2	—	—	—
AS	Nepal (NE)	582	NA	33	41	26	—	—	—
EU	Austria (AU)	7956	7528	58	39	3	49	27	24
EU	Belgium (BE)	492	348	41	24	35	39	23	38
EU	Bulgaria (BU)	684	180	58	35	8	60	30	10
EU	Croatia (CR)	218	NA	26	34	40	—	—	—
EU	Cyprus (CY)	128	NA	59	38	2	—	—	—
EU	Czechia (CZ)	913	170	44	31	25	47	22	32
EU	Denmark (DE)	5633	2287	58	33	9	28	11	61
EU	Estonia (ES)	245	248	75	21	4	35	56	9
EU	Finland (FI)	455	478	85	13	2	49	32	18
EU	France (FR)	3799	1960	72.5	19.9	7.6	48	29	23
EU	Germany (GE)	8101	5915	27.5	35.8	36.6	20.3	35	44
EU	Greece (GR)	321	105	42.4	37.7	19.9	42.9	25	32
EU	Hungary (HU)	791	446	39.4	39.6	21.0	27.8	38	35
EU	Ireland (IR)	2341	175	59.1	23.3	17.6	56.0	37	7
EU	Italy (IT)	2832	235	62.0	24.0	14.0	80.0	16	4
EU	Lithuania (LI)	480	385	72.7	24.0	3.3	39.2	30	31
EU	Luxembourg (LU)	106	45	50.0	28.3	21.7	42.2	22	36
EU	Latvia (LA)	179	100	41.9	44.1	14.0	35.0	41	24
EU	Netherlands (NE)	239	232	17.2	54.8	28.0	14.7	29	57
EU	Norway (NO)	1876	1791	29.7	31.4	38.9	10.0	49	46
EU	Poland (PO)	3507	3065	56.3	37.8	5.9	58.9	38	3
EU	Portugal (PT)	876	143	67.5	20.3	12.2	34.3	18	48
EU	Romania (RO)	2543	894	91.3	8.5	0.1	92.7	7	1
EU	Slovakia (SL)	313	213	51.4	35.1	13.4	62.9	26	11
EU	Slovenia (SLO)	134	4	67.2	26.1	6.7	100.0	0	0
EU	Spain (SP)	3147	367	75.5	14.9	9.6	58.9	25	17
EU	Sweden (SW)	1495	2487	66.1	29.6	4.3	43.1	39	18
EU	United Kingdom (UK)	6814	4881	80.1	13.9	6.1	69.4	16	15
NA	Coastal Plains—USA (COP)	218	205	14.0	22.0	64.0	17.0	19	64
NA	Northern Appalachians—USA (NAP)	252	242	40.0	23.0	37.0	44.8	12	44
NA	Southern Appalachians—USA (SAP)	248	208	23.2	29.3	47.5	27.7	39	34
NA	Upper Midwest—USA (UM)	159	142	39.0	31.0	30.0	42.7	23	35
NA	Temperate Plains—USA (TP)	219	204	24.0	30.0	46.0	30.1	33	37
NA	Northern Plains—USA (NP)	172	136	50.0	12.0	38.0	44.3	25	30
NA	Southern Plains—USA (SP)	133	118	33.0	41.0	26.0	18.0	42	40
NA	Western Mountains—USA (WM)	263	181	51.5	18.2	30.3	38.2	16	46
NA	Xeric—USA (XE)	183	121	22.0	34.0	44.0	28.8	30	41
NA	Central catchments—Mexico (CM)	48	30	28.0	10.0	62.0	23.0	14	63
NA	Western coastal catchments—Mexico (WCM)	16	39	69.0	12.0	19.0	51.0	36	13
NA	Newfoundland—Canada (NF)	137	NA	49.0	51.0	0.0	—	—	—
NA	British Columbia—Columbia basin—Canada (CO)	305	NA	75.0	10.0	15.0	—	—	—
NA	British Columbia—Okanagan\Similkameen basins—Canada (OK)	138	NA	65	11	24	—	—	—
NA	British Columbia—Fraser basin—Canada (FRA)	192	NA	62	21	17	—	—	—
NA	British Columbia—North Coastal catchments—Canada (NC)	327	NA	92	6	2	—	—	—
NA	British Columbia—Peace basin—Canada (PE)	128	NA	80	10	10	—	—	—
O	New Zealand (NZ)	995	NA	38	38	24	—	—	—
O	Murray–Darling basin—Australia (MD)	1568	513	50	42	8	9	35	56
O	Southeast Coast—Australia (SEC)	1687	NA	48	41	11	—	—	—
O	Southwest Coast—Australia (SWC)	183	NA	20	45	36	—	—	—
O	Tasmania—Australia (TA)	2286	NA	62	29	9	—	—	—
SA	Brazil‐Cerrado BR‐C)	269	217	34	35	31	25	63	12
SA	Brazil‐Amazon (BR‐A)	92	92	42	58	0	28	60	12
SA	Bolivia ‐ Tropical rainforest (BO‐T)	56	0	57	36	7	—	—	—
SA	Bolivia‐Andean mountains (BO‐M)	143	0	38	46	16	—	—	—

*Note*: NA, data not available. Africa = AF, Asia = AS, Europe = EU, North America = NA, South America = SA, Oceania = O. Data sources in Table S1.

### 2.2 Biological data

We targeted the most recent bioassessment data available for each site, present in national/continental validated databases or from peer‐reviewed publications, and with validated and/or published biological classification methods (Tables S1 and S2). We collected benthic macroinvertebrate and fish assessment data for the same regions whenever data from both elements were available. Most assessments were based on ad hoc or disturbance‐gradient site selection; data from Brazil‐Cerrado, the USA, and of fish from the Australia's Murray–Darling Basin were based on probability sampling designs. Because a site on a small stream represents a much greater percentage of stream length than a site on a large river, quality assessments are reported as percentage of stream length throughout this paper.

Benthic macroinvertebrates were sampled in most countries with a hand‐net by kick and sweep sampling or with a Surber sampler (Japan, Nigeria, South Africa, South Korea); and with grabs in non‐wadeable rivers (South Korea, Lower Mekong River). The sampling procedure was multi‐habitat or included only hard substrates (Sweden). Fish usually were sampled by multi‐habitat electrofishing across the entire site. Cast‐nets or seines and hand nets were used in addition to electrofishing in some countries (Japan, Mexico, Brazil) or alone (South Korea). For sites that were sampled multiple times, we only used results from the most recent visit.

The biological quality indices used were those previously validated for the study regions and were based on the reference condition approach (RCA, Reynoldson et al., 1997; Stoddard et al., 2006; Table S2). This means that the index scores were adjusted to the reference values of the most appropriate river type/ecoregion. For multimetric indices and predictive models the index score produced is on a 0–1 scale. The use of the RCA and normalized scales contributes to reducing the importance of different sampling methods and indices.

Most of the indices used for benthic macroinvertebrates (Table S2) were traditional or predictive multimetric (MMI), including in Europe (e.g., Portugal, Italy, Belgium, Spain), Brazil—Cerrado (Silva et al., 2017), Mexico (Pérez‐Munguía et al., 2005), Nigeria (Edegbene, 2020), Bolivia (Moya et al., 2011), and the United States (Herlihy et al., 2020; Stoddard et al., 2008). Some are biotic indices based on organic pollution tolerance (e.g., Hungary, Croatia, Estonia, New Zealand—Stark, 1985, Dickens & Graham, 2002, Nepal—Tachamo‐Shah & Shah, 2012, South Korea—Kong et al., 2018), which have been used to detect an array of other river alterations (e.g., Leps et al., 2015). Other indices were predictive taxonomic richness models (UK‐ Kral et al., 2017, Australia—Davies, 2000, Canada—Reynoldson et al., 1997, China—Chen et al., 2019). Japan used EPT genus richness, and the Amazonian sites were assessed with TITAN (threshold indicator taxa analysis, Martins et al., 2021). The 27 European indices developed or adopted in the context of the implementation of the Water Framework Directive (EC, 2000) were subjected to intercalibration exercises where the quality‐class boundaries were checked against each other and adjusted if needed to guarantee comparable assessments (Birk et al., 2013; Feio et al., 2014; Poikaine et al., 2014) and published, along with the adopted class‐boundaries (Commission Decision, 2018; Feio, Hughes, et al., 2021).

Traditional or predictive MMIs were also most commonly adopted for fish assemblage assessments. For fish in Europe (Table S2), MMIs were adopted in most countries (Commission Decision, 2018) and there was also an intercalibration exercise (Birk et al., 2018; Jepsen & Pont, 2015). In the United Kingdom and France, predictive MMIs were used (Commission Decisions, 2018; Feio, Hughes, et al., 2021). Fish MMIs were also used in New Zealand (Joy & Death, 2004), USA (USEPA, 2020), Mexico (Lyons et al., 1995), Brazil (Carvalho et al., 2017), South Korea (ME 2016), and Australia (Davies et al., 2010; Lintermans et al., 2005). Japan used native freshwater fish species richness. Amazonian sites were assessed with TITAN (Martins et al., 2021).

To homogenize site assessments globally and reduce the importance of differences in study designs and sampling and assessment methods, we converted all the site quality classification systems into a simple three‐class system: Good (no rehabilitation measures needed, assemblages similar to reference conditions); Impaired (scores clearly lower than in reference conditions); and Severely Impaired (many fewer taxa than expected; substantial alteration in assemblage composition compared to reference conditions). In practical terms, this means that the High and Good classes from European indices, the Equivalent to Reference of BEAST predictive models, the A band of AUSRIVAS and RIVPACS models, or classes A (Pristine) and B (largely natural) of the South African system were considered as Good in our classification system. The remaining degradation gradient was divided into two classes. An intermediate degradation class (Impaired) corresponds with classes C (moderately modified) and D (largely modified) in the South African system, and to the Moderate and Poor classes of the WFD system. A substantial degradation level (Severely Impaired) integrated classes E (seriously modified) and F (Critically modified) from South Africa, and Bad from the WFD. Those results were reported as % of sites in Good, Impaired, and Severely Impaired for each study region.

### 2.3 Global climate characterization

The study regions (Table 1) were characterized regarding their climate using the Köppen‐Geiger main climate type (Equatorial, Arid, Warm, Snow, or Polar) based on projections for the period 2001–2025. The metadata were extracted from http://koeppen‐geiger.vu‐wien.ac.at/shifts.htm. When more than one climate type was present in a study region the spatially most extensive one was chosen.

### 2.4 Global anthropogenic pressure and ecosystem protection indices

To demonstrate the associations of anthropogenic pressures with biological conditions in a consistent manner, we correlated site biological quality against five global landscape indices. As a measure of land use alteration from human activities, we used the Human Footprint index (HF; Human Footprint 2004; WCS & CIESIM, 2005). The HF integrates data on population density, land transformation and infrastructure and human accesses (roads, railways and navigable waterways). It is expressed in percentage of relative human influence in each terrestrial biome and is divided into eight categories. This index integrates HF observations from 1995 to 2004 and was determined for each study area. Although we used biological data from 2001 to 2020, we used the HF index as a static measure of relative anthropogenic pressures on aquatic ecosystems that we assume extends into 2020. We also used the HDI scores extracted from the data center (http://hdr.undp.org/en/content/human‐development‐index‐hdi) for the period 2000–2019 as a measure of social well‐being in each country. This index includes information on life expectancy at birth, expected years of schooling, mean years of schooling, and gross national income per capita. Finally, for each country, we obtained the values of three indicators associated with the United Nations Sustainable Development Goal (UN SDG) 6 from its Agenda 2030 (UN, 2015). (1) The “% of rivers with good ambient water quality” relies on water quality data derived from in situ measurements and the analysis of samples collected from surface and groundwaters. (2) The “% of protected freshwater key biodiversity areas (KBAs)” is the average proportion of freshwater KBAs covered by protected areas. (3) The “% of forest area net change rate” is based on the sum of all changes in forest area including reductions from deforestation and disasters and increases from afforestation and expansion of forests during the period 2010–2020.

### 2.5 Data analyses

We used PERMANOVA (Permutational Analysis of Variance; main and pairwise tests; PRIMER v7; Clarke & Gorley, 2015) to determine if there were significant relationships in the biological classifications among sites with different climate types. Euclidean distance was used as a similarity measure. Lin's concordance correlation coefficient (Lin, 1989) and Pearson's product–moment correlation coefficient (*r*; Rovine & Von Eye, 1997) were used to test the correlation and agreement between fish and macroinvertebrate classifications. Pearson's *r* is a measure of the strength of a linear association between two variables. Pearson correlation was also used to test for significant correlations between the values of the global indices (HF, HDI, % of rivers with good ambient water quality, % of protected freshwater KBAs, % forest area annual net change rate) and macroinvertebrate and fish classifications, independently. Data were a priori checked for normality with Shapiro–Wilk tests (Shapiro & Wilk, 1965) and for homoscedasticity with studentized Breusch–Pagan tests (Breusch & Pagan, 1979). Data were approximated to normal distribution through arcsine transformations.

We performed the statistical analyses (with the exception of the PERMANOVA) in R (R Core Team, 2021). For parametric assumptions, we used the following: (1) Shapiro.test function for normality (R Stats, R Core Team, 2021), visualized using the ggqqplot function (ggpubr vs 0.4.0 package; Kassambara, 2020) and (2) bptest function for homoscedasticity (lmtest package, Zeilieis & Hothorn, 2002). For correlations we used cor.test function (R Stats, R Core Team, 2021); and agree.ccc for the Concordance Correlation Coefficients (agRee vs 0.5‐3 package, Feng, 2020).

For the global biological quality maps we used vectors of World Administrative Boundaries (https://public.opendatasoft.com/explore/dataset/world‐administrative‐boundaries/export/), FAO continental map of major hydrological basins (https://data.review.fao.org/map/catalog/srv/eng/catalog.search#/metadata/7707086d‐af3c‐41cc‐8aa5‐323d8609b2d1), USEPA ecoregions (https://www.epa.gov/national‐aquatic‐resource‐surveys/ecoregions‐used‐national‐aquatic‐resource‐surveys), and ESRI ArcGis software for constructing the final maps.

## 3 RESULTS

### 3.1 Coverage of the sampling sites obtained

We obtained biological quality assessments for 72,275 river sites based on macroinvertebrate assemblages and 37,676 based on fish assemblages, collected between 2001 and 2020 (Table 1). In the Northern Hemisphere, the data sets consistently covered the European continent and the conterminous United States. For Canada, invertebrate data were available only for portions of the provinces of British Columbia and Newfoundland. There were no appropriate fish data available for Canada. From North America, we also included Mexican data from 64 macroinvertebrate sites and 69 fish sites.

In South America, the country with the largest number of sites in this study was Brazil, where both macroinvertebrate and fish data were gathered from the same sites (361 macroinvertebrate sites, 317 fish sites). Those data were obtained from two large 2010–2019 research studies covering two different biomes, the Brazilian savanna (Cerrado) and the Amazon rainforest. We also included a Bolivian data set (199 invertebrate sites) covering montane (Andean mountains) and tropical rainforest areas. Neither data set covered all of Bolivia or Brazil nor all the local biomes.

From Africa, our data were limited to South Africa and Nigeria. In South Africa, there is a good representation of catchments with 1426 sites obtained in official monitoring programs for macroinvertebrates. No recent fish data were available. In Nigeria, the representation is limited to 69 sites sampled for recent research projects.

From Asia, we gathered data from eight countries, most of which are underrepresented in riverine bioassessment scientific studies published in English. For Japan, we acquired data for 563 sites (macroinvertebrates) and 547 sites (for fish) collected via several government programs from 2001 to 2006. South Korea, also with official programs covering all catchments, produced data for 1154 sites (macroinvertebrates) and 1156 sites (fish), all sampled in 2019. Nepal was represented in our data set by 582 macroinvertebrate sites sampled in both the Eastern and Western Himalaya between 2010 and 2021. We included 41 macroinvertebrate sites sampled in 2017 from the lower Mekong River, in Laos, Thailand, Cambodia and Vietnam through an international cooperation project (Mekong River Commission). Invertebrate data were also included from China, with the largest data set in Asia of 1405 sites distributed across Upper Mekong (176 sites), Yangtze basin (483 sites), Zhu basin (262 sites), and Zhejiang coastal (484 sites) and sampled from 2004 to 2020. These data originated from different research projects and are being used to develop common predictive models (Chen et al., 2019, Chen et al. unpublished). However, no fish data were available for the Asian nations other than Japan and South Korea.

In Oceania, the largest data set covers the Australian Murray–Darling Basin, and the large hydrographic regions of Southeast Coast, Southwest Coast and Tasmania (5724 sites for macroinvertebrates). For fish only the Murray–Darling Basin was covered (513 sites). In New Zealand, there is a good representation of rivers in the 995 sites of macroinvertebrates sampled in 2019. Recent fish data were not available.

### 3.2 Biological quality of river sites

Globally, 50% of the sites assessed were in good condition according to benthic macroinvertebrates, 29% were Impaired, and 21% were Severely Impaired (Table 1; data available at: https://doi.org/10.5061/dryad.pnvx0k6r9). The assessments based on fish showed slightly greater degradation with only 42% of the sites in Good condition, 29% Impaired and 29% Severely Impaired. For the same study regions with both benthic macroinvertebrate and fish data, site‐quality classifications based on fish were consistently lower (ca. 7% average difference), with few exceptions (i.e., USA, Finland; Table 1). Despite the differences, the biological condition of river sites based on the two biological elements were moderately correlated and concordant (Pearson *r* = 0.62, *p* < .001, *df* = 40 and 0.32 < Lin's *R* = 0.58 < 0.76 for Good quality; *r* = 0.47, *p* = .002, *df* = 40 and 0.13 < Lin's *R* = 0.46 < 0.70 for Impaired; and *r* = 0.63, *p* < .001, *df* = 40 and 0.56 < Lin's *R* = 0.56 < 0.79 for Severely Impaired sites; Table S3; Figure S1).

**FIGURE 1:**
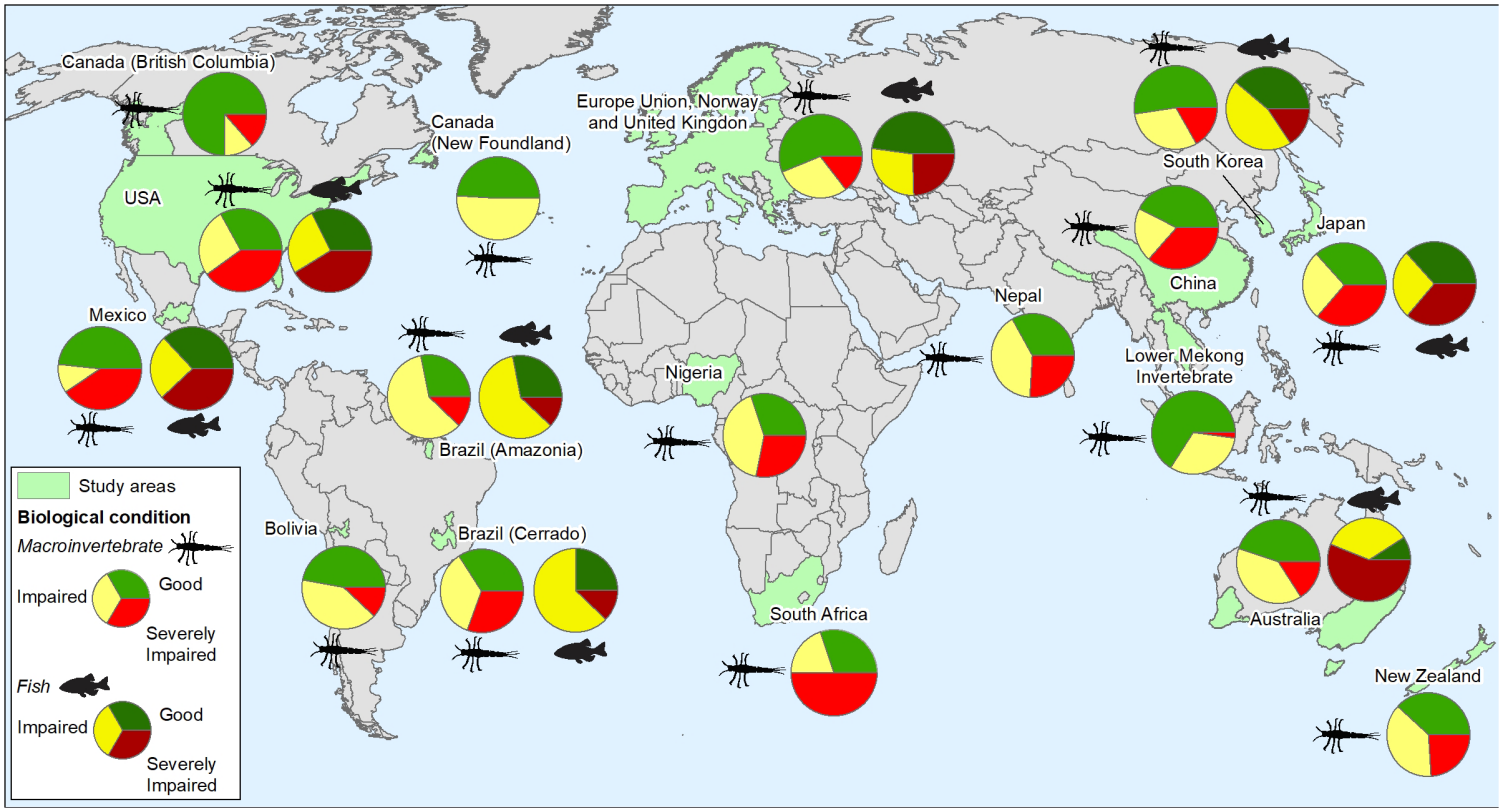
Percent of sites in Good (green), Impaired (yellow), and Severely Impaired (red) condition based on benthic macroinvertebrate and fish assemblages by large regions. Map lines delineate study areas and do not necessarily depict accepted national boundaries.

**FIGURE 2:**
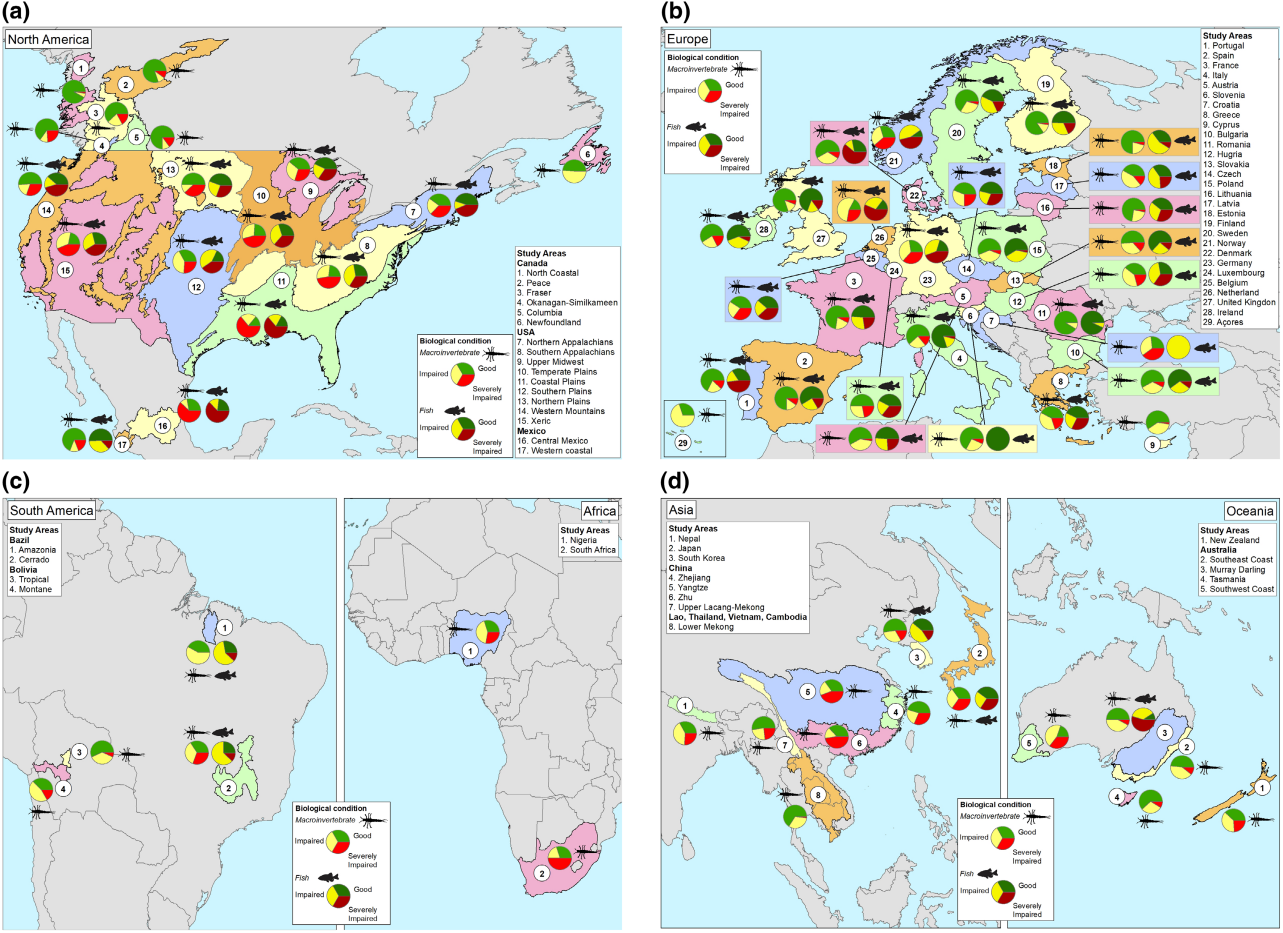
Location of the study regions by continent with the percent of sites in Good (green), Impaired (yellow), and Severely Impaired (red) condition by study region (see Table 1), based on benthic macroinvertebrates and fish. Map lines delineate study areas and do not necessarily depict accepted national boundaries.

Considering macroinvertebrate condition at the continental level or large continuous regions, 75% of the sites in some European countries (Finland, Spain, Romania, Estonia, UK) and British Columbia (Canada) were in Good condition, followed by the Lower Mekong River (Asia, 66%), Europe (56%), and South Korea (53%). Regions with a third or fewer of the sites in Good condition occurred in South Africa (30%), Nigeria (30%), and the conterminous United States (33% of stream length). Less than 25% of the sites were in Good condition in The Netherlands (Europe), US Coastal Plains, and central Mexico (North America) (Figure 2a,b). Higher percentages of Severely Impaired sites were found in South Africa (50%) and the conterminous United States (40% of stream length), but only 0 to 2% of sampled sites in Amazonia, Newfoundland, and the Lower Mekong River were Severely Impaired (Figures 1 and 2a,c,d).

For fish assemblages, there was no region with at least half the sites in Good condition. The best conditions were found in European countries (48% Good on average), South Korea (39%), and Japan (40%). The regions with the worst quality sites based on fish assemblages were in the Murray–Darling Basin (Australia, 56% Severely Impaired) and the United States (on average, 41% Severely Impaired; Figure 1). But there were also countries within Europe with a high proportion (>45%) of Severely Impaired sites for fish (e.g., Denmark, Norway, The Netherlands, Portugal) (Figure 2b).

**FIGURE 3:**
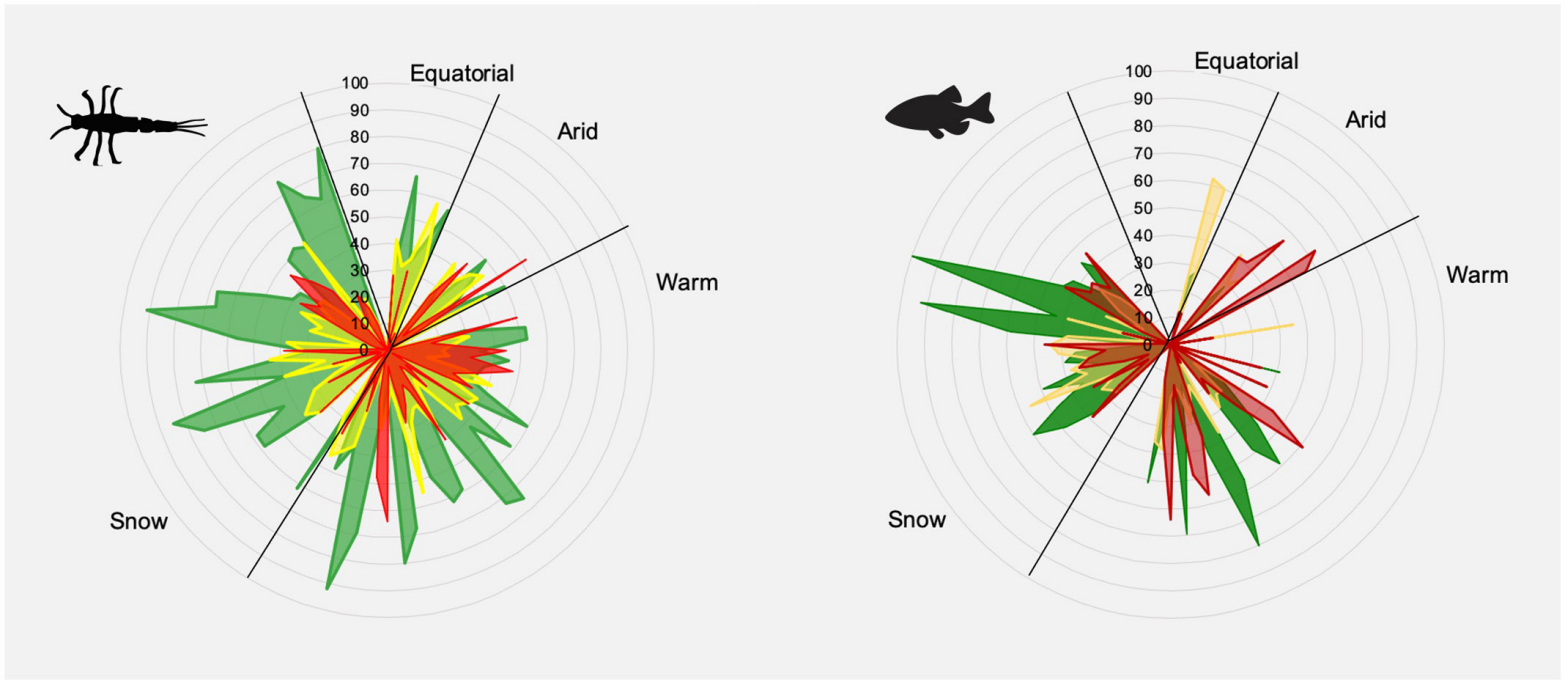
Distribution of sites (percent) in Good (green), Impaired (yellow), and Severely Impaired (orange) condition based on benthic macroinvertebrates (left) and fish (right) by climate type (A = Equatorial, B = Arid, C = Warm, D = Snow).

There were significant differences among major climate types in terms of biological condition (Figure 3; PERMANOVA main tests: macroinvertebrates—*Pseudo‐F* = 2.433, *p* = .031, *df* = 3; fish—*Pseudo‐F* = 4.530, *p* = .002, *df* = 3). For macroinvertebrates, significant differences occurred between Arid and Snow climate types (paired *t*‐test: *t* = 2.122, *p* = .017). Significant differences occurred for fish between Equatorial and Arid (*t* = 3.950, *p* = .046), Equatorial and Warm (*t* = 2.146, *p* = .021), Equatorial and Snow (*t* = 1.940, *p* = .032), Arid and Warm (*t* = 2.387, *p* = .017), and Arid and Snow (*t* = 2.978, *p* = .001) climate types. The highest percentages of sites in Good condition were found in Snow climate (56% for macroinvertebrates, 47% for fish), the highest percentages of Impaired sites were in the Equatorial climate (41% for macroinvertebrates, 61% for fish), and the highest percentages of Severely Impaired sites were in the Arid climate (27% for macroinvertebrates and 51% for fish).

### 3.3 Effects of human activities on riverine biological quality

The percent of Impaired fish sites was negatively correlated with HF (Pearson *r* = −0.42, *p* = .006, *df* = 40) and the percent of Severely Impaired fish sites was positively correlated with the HDI (Pearson *r* = 0.37, *p* = .015, *df* = 40; Table S3; Figures S2 and S3). The condition of river sites based on macroinvertebrates was not significantly correlated with any of the indices.

The biological quality classifications were also significantly correlated with UNSDG 6 indicators (Table S3; Figures S4–S6). The percent of sites with Good ambient water quality were positively correlated with that of sites in Good condition based on macroinvertebrates (*r* = 0.43, *p* = .003, *df* = 45) and fish (*r* = 0.30, *p* = .084, *df* = 33), and negatively correlated with the percent of Severely Impaired sites based on macroinvertebrates (*r* = −0.52, *p* < .001, *df* = 45) and fish (*r* = −0.36, *p* = .032, *df* = 33). The percent of sites in Good condition based on fish was positively correlated with the percent of forest area net change rate (*r* = 0.30, *p* = .058, *df* = 40), and the percentages of Impaired sites based on macroinvertebrates (*r* = −0.26, *p* = .034, *df* = 62) and fish (*r* = −0.28, *p* = .074, *df* = 40) were negatively correlated with the same index. Finally, the percent of Severely Impaired sites based on macroinvertebrates was negatively correlated with the percent of protected freshwater KBAs (*r* = −0.31, *p* = .011, *df* = 62). The *r* values, degrees of freedom, and *p* values (Table S3) follow the APA recommendations for this type of statistical analyses.

## 4 DISCUSSION

Only half of the assessed sites included in this study were in Good biological condition and almost a third were Severely Impaired. This is consistent with studies on losses of freshwater vertebrate biodiversity (e.g., Albert et al., 2021; Darwall et al., 2018; Dirzo et al., 2014). Additionally, we found that river condition assessed by using fish assemblages was typically lower than the condition based on macroinvertebrates (e.g., 20%–40% difference in Europe). Because both fish and macroinvertebrates are affected by changes in water quality and forest area (especially in the riparian zone), this difference may be driven by the hydromorphological alterations of rivers. Fish move across larger spatial scales and across habitats within rivers than invertebrates to access food resources and complete their life cycles (O'Mara et al., 2021). Therefore, they are severely affected by losses in longitudinal connectivity caused by the widespread fragmentation of rivers and streams by dams, reservoirs, and poorly constructed road‐stream crossings for at least three reasons: (1) Fish cannot pass physical barriers (as can the flying adult stages of many aquatic insects). (2) Reservoirs are often associated with increased abundances of invasive non‐native fish (Clavero & Hermoso, 2011; Jellyman & Harding, 2012). Although invasive invertebrates are also a concern in reservoirs (e.g., Jovem‐Azevedo et al., 2022), comparatively more studies indicate a catastrophic effect of invasive non‐native fish on native assemblages (Leprieur et al., 2008; Pelicice & Agostinho, 2009; Rinne et al., 2005; Weyl & Ellender, 2014). (3) Species in several fish families (e.g., lampreys, salmonids, eels, herrings, sturgeon, catfishes, characids) have life histories requiring long‐distance migrations (Brönmark et al., 2013; Oberdorff & Hughes, 1992), whereas most macroinvertebrates are relatively sedentary (Bilton et al., 2001). In agreement, Belletti et al. (2020) reported the existence of at least 1.2 million instream barriers in 36 European countries and Grill et al. (2019) estimated that 63% of the world's large rivers are no longer free‐flowing (based on a connectivity status index, that includes but not exclusively longitudinal connectivity). Indeed, this may be one of the reasons for the poor conditions of fish assemblages in the Murray–Darling Basin, Australia (56% Severely Impaired), with >4000 instream barriers (Lintermans, 2007) and in the United States (41% Severely Impaired), where dams were significant factors for low MMI scores in the Coastal Plains and Southern Appalachian Plateau ecoregions (Herlihy et al., 2020). In addition, >45% of the sites were Impaired based on fish assemblages in Denmark, Norway, The Netherlands, central Mexico, and Portugal (all of which have relatively high densities of impassable dams; Belletti et al., 2020; Mercado‐Silva et al., 2008; Soininen et al., 2017). These results for fish assemblages plus their moderate correlations with macroinvertebrate quality values (Table S2; Figure S1) indicate the importance of including both fish and macroinvertebrate assemblages in rigorous riverine bioassessments (Herlihy et al., 2020).

The European Commission also recognized that hydromorphological pressures are one of the main reasons for not reaching the good ecological status of water bodies (the minimum acceptable quality according to the WFD) and aims to return at least 25,000 km of rivers into free‐flowing status by 2030 (EEA, 2021). Recent studies in temperate regions reinforce this urgency, as climate changes in addition to damming are expected to cause major alterations in riverine biological communities, including macroinvertebrate, macrophyte and microalgae assemblages, ranging from decreases of 76% to increases of 67% in abundance/cover in regulated rivers by 2050 during summer months (Rivaes et al., 2022).

Overall, our results do not support our first hypothesis that countries with a longer history of human settlements and modifications (disregarding the impact of First‐Nations) would have a greater proportion of sites in poorer condition. For example, some regions of the United States or Brazil have lower percentages of Good quality sites than many European countries, with a much longer history of non–First Nations land management. Yet, we observed substantial differences in the biological quality of rivers associated with the social development state of countries. Most regions with a high HDI in the last two decades (e.g., in Northern and Central Europe, Canada, and Australia), had a lower percentage of impaired sites than other regions of the world. This could result from either of two possible reasons. (1) The Canadian regions have low population densities and extensive preserved areas. (2) Those regions have recently implemented strong environmental protection measures and ecosystem rehabilitation that may have facilitated some recovery of rivers (Feio, Hughes, et al., 2021). This is consistent with the negative correlations of the HF index and the proportions of Impaired and Severely Impaired sites but also with recent improvements of the HF for these countries (Venter et al., 2016). However, the United States is an exception to this pattern, with <25% of the sites in Good condition in the Coastal Plains (based on macroinvertebrates) and 40% of sites being in Severely Impaired condition for the conterminous United States. This is largely driven by extensive and intensive agriculture and livestock grazing pressures (Hughes & Vadas Jr., 2021). The best predictors of poor (Severely Impaired) MMI scores in logistic‐regression, risk‐assessment models were excess nutrients, turbidity, excess fine sediments, and dams (Herlihy et al., 2020). Nonetheless, the greater implementation of freshwater protection areas was associated with a lower proportion of Severely Impaired river conditions in the United States.

The higher protection of rivers could also be one of the reasons for a better condition of rivers in Snow climates, which are represented in our data sets by countries of northern Europe, Canada and partially in northern United States. Conversely, poorer condition was found in Arid and Equatorial areas, corresponding in our data to sites located in South America, Nigeria, Mexico, the United States (Xeric and Southern Plains), and Australia (Murray–Darling Basin). In Arid regions, the poorer biological quality may be also the result of greater societal demands for surface and groundwater, in combination with other pressures, which disrupt flows and ecosystem functioning (Chen & Olden, 2017; Kattel, 2019).

Despite the potential synergistic influence of climate, in Africa, Asia, and South America, the biological quality results tend to track the physico‐chemical water quality global conditions (Mekonnen & Hoekstra, 2017 *)*. In South Africa and Nigeria, a third of the sites were in good condition based on macroinvertebrate assemblages. In South Africa these results can be attributed to increasing urbanization and the associated poor wastewater treatment works (DWS, 2016). In Nigeria, enforcement of environmental laws is weak and therefore river degradation especially in urban areas is common.

The Chinese basins of the Yangtze River and Zhu River had only one‐third of sites in Good condition based on macroinvertebrate. These basins have undergone vast changes from centuries of human occupation resulting in river fragmentation and flow regulation, water pollution by industrial and domestic sewage discharges, logging and sand mining (Jin et al., 2022; Liu et al., 2013; Zhang et al., 2010). In contrast, the Upper Mekong and Zhejiang coastal sites, with nearly one‐half of sites in Good quality, have their greatest forest cover in 2010 and lowest forest loss between 2001 and 2010 (Chen et al., unpublished data). In Japan water pollution is also declining; however, fragmentation of river networks, decreases of flow and sediment dynamics, river channel and bank stabilization, degradation of floodplains, and invasive non‐native species have contributed to the declines in fish and benthic macroinvertebrate diversity (Akasaka et al., 2022; Nakamura et al., 2006; Nakamura et al., 2017). A better scenario was found in the Lower Mekong River, where 66% of the sites were in Good condition for macroinvertebrates. Indeed, the water quality there is generally good, which is not surprising as there are fewer large cities and industry or industrial agriculture compared to other regions in Asia (MRC, 2019). In addition, the two largest cities (Vientiane and Phnom Penh) are located on natural levees, and their drainage/sewage passes through wetlands before reaching the river at least 10 km downstream of the study sites. Nonetheless, dam building on the Mekong is a major threat to fisheries, biodiversity, and other ecosystem services (Winemiller et al., 2016).

In Brazil, agriculture is a major pressure on the small streams in the study regions (Couto et al., 2021). The Amazonian region, where a large proportion of the study catchments were still intact (although suffering from recent deforestation; Pelicice & Castello, 2021), has the lowest percentages of Severely Impaired sites (none based on macroinvertebrates and 12% for fish). This shows the importance of maintaining well‐preserved areas; however, the high percentage of Impaired sites (58%–60%) indicates increased forest clearing for agriculture as shown in structural equation modeling (Leitão et al., 2018). In the Cerrado region, the results show greater degradation, which was significantly associated with intensive agriculture and pasture in risk assessment models (Martins et al., 2021; Silva et al., 2018). Moreover, untreated domestic sewage in urban areas is still an important source of degradation (Carvalho et al., 2017), favoring more resistant non‐native species to the detriment of native fishes (Carvalho et al., 2019). Indeed, 40% of Brazilian cities and 100 million citizens have no sewage collection or treatment and the New Regulatory Framework for Basic Sanitation (Law Project 4162/2019) assumes the goal of water supply and sewage treatment by 2033 (Callisto et al., 2021). Similarly, many rivers in central Mexico suffer from declines in water quality and desiccation which contribute to macroinvertebrate and fish population fragmentation and lead to lower MMI values (Mathuriau et al., 2011).

Our results reflect those of Romero et al. (2021) who reported declines in abundance and diversity of all major orders and families of sub‐tropical freshwater insects in Brazil over 20 years. Regions that typically support high biodiversity, such as Brazil, South Asia and Central Africa, have seen a rapid increase in their HFs (Venter et al., 2016), which also endangers river ecosystems. The decline in biodiversity is expected to be aggravated by the large hydropower dams and water‐transfer schemes planned or under‐construction in Asia, Brazil, India, and Nepal (Grill et al., 2019) that are also responsible for the introduction of non‐native species (Bueno et al., 2021), and by water scarcity in the driest regions which is expected to worsen with climate change (Tauro, 2021, Winfield et al., 2016).

Our study also showed that alterations in terrestrial ecosystems, particularly forests, were associated with the biological condition of rivers indicated by macroinvertebrate and fish assemblages. The negative correlation of benthic macroinvertebrate condition with the decrease in forests is expected because many species depend on allochthonous organic matter (leaves, fruits, wood) provided by surrounding forests as energy sources (e.g., Boyero et al., 2020; Jonsson & Sponseller, 2021; Silva‐Araújo et al., 2020). Moreover, forests surrounding rivers constitute efficient buffers against pollution caused by other land uses, such as agriculture, because the filtering capacity of the vegetation improves water quality, which influences aquatic communities (Aguiar et al., 2011; Effert‐Fanta et al., 2019; Riis et al., 2020). In Japan, for example, the higher richness of red‐listed fish species was associated with high percentages of forest cover in the catchments (Lavergne et al., 2021). Because the highest rates of forest loss were identified in important biodiversity regions, such as in Bolivia, Brazil, Nigeria and the Lower‐Mekong River basin, we can expect an increasing global rate and a net biodiversity loss in freshwaters (Leal et al., 2020).

Despite the large number of sites included in this study, our results are biased by insufficient information from large continents (Africa, Asia, South America) and large countries (e.g., Russia, Canada, Brazil, Indonesia, India, Democratic Republic of Congo, Algeria, and Argentina). Our data were also spatially restricted in Australia, Bolivia, Brazil, Cambodia, China, Laos, Mexico, Nepal, Thailand, and Vietnam, so national assessments of those nations' rivers were impossible as well. In addition, biological assessment data for many countries was lacking or existing studies failed to meet our criteria for sampling sites to cover a large region/basin, having already well‐established indices adapted to local conditions and following RCA, and quality classification systems for macroinvertebrates and/or fish (see Materials and Methods; Feio, Hughes, et al., 2021, Zhang et al., 2021). This lack of information limits our global conclusions and often results from insufficient concern of governments for aquatic bioassessments and water resource conservation or too few resources to implement such programs.

The implementation of statutory bioassessment programs was indeed, the reason for the high coverage of some regions and countries with bioassessment data, such as in Europe, Japan, New Zealand, South Korea, and the United States and should thus be continued. This approach is preferable against independent monitoring programs or the use of research data not specifically collected for assessment purposes, which makes comparisons difficult across regions and provides limited ability for continuity and large spatial coverage of river basins. Rivers often span political boundaries, and their condition is best protected and managed under national or multi‐national policies supported by a coordinated bioassessment framework (Nichols et al., 2017; USEPA, 2020). On the other hand, the interruption of national programs in large countries such as Canada and Australia, and from which we gathered data for this study, is worrying as data discontinuity hinders future assessment of how implemented management practices alleviate or worsen the biological condition of rivers. Other issues that potentially influenced our results are the differences in sampling designs, sampling methods, indices and classification systems across the regions studied (Table S2). In many nations, river assessments are biased towards sampling wadeable streams versus boatable rivers because of the greater logistical costs of sampling large rivers. However, small systems also represent most of the river length in an area (Colvin et al., 2019). The use of the RCA means that the sampled sites are assessed against the best‐available or least‐disturbed conditions for a given region or type of river (EC, 2000; Herlihy et al., 2008; Stoddard et al., 2006). However, there are national and regional biases amongst reference sites depending on river size and the extent and intensity of landscape disturbances (Stoddard et al., 2006). Also, some sampling programs may be biased toward assessing more degraded sites and ad hoc site selection precludes inferring site results to all waters of entire regions (Downs, 2010; Hughes et al., 2000). Therefore, our results pertain only to the sampled sites except in surveys based on probability designs (fish of Australia's Murray–Darling Basin, Cerrado, USA) or where the characterization of all waterbodies was targeted and thousands of sites are sampled (as in European programs designed for the implementation of the Water Framework Directive).

There are however several reasons for greater confidence in our comparisons and the following four steps mitigated the potential influences of differing study designs, reference conditions, sampling methods, and indices. (1) The assemblage composition (taxa × abundance), was not compared nor the scores of the indices, but used only the quality‐class assessments. The influence of such differences in methods is minimized when using indices and quality classes instead of assemblage composition (Herbst & Sildorff, 2006; Houston et al., 2022). However, methods such as EPT and fish species richness used in Japan and TITAN in the Amazon failed to do this. (2) In the European Union, the member‐states followed standard procedures according to the WFD and an intercalibration process was run to minimize potential boundary differences among quality classes of different indices (e.g., Birk et al., 2013; Feio et al., 2014; Poikaine et al., 2014). (3) The numbers of sites in each classification were not compared. Instead, the comparison was made among the percent of classifications obtained in each region for each class. (4) As specified in the methods, the original classifications of indices were not compared. Instead, we normalized all the classifications into a simpler three‐class system. Instructions were given to all authors on how to define each of these three classes. Yet, the interpretation of what should be attributed to each of the three classes may have some subjectivity, especially regarding the differences between Impaired and Severely Impaired sites that may cause some bias in our results. But overall, the advantage of combining the data sets overcomes the potential drawbacks as is allows a comparison across a very wide range of countries, including those not previously compared.

Finally, our correlations between the bioassessment results and the global disturbance and SDG indices, although providing interesting insights, are weaker than the ideal because of the different extents of assessments. For example, the HDI is reported nationally, but there are substantial regional differences in larger countries. In addition, there were temporal mismatches between the global indicators and the river biotic indices, although we chose the most appropriate periods to the degree possible.

### 4.1 Implications of poor global biological quality and recommendations for river management

The poor biological quality of rivers in the study regions reflects a global loss of freshwater biodiversity, altered species distributions, simplification of aquatic community structure and composition, and increased invasive non‐native species. Such changes disrupt ecosystem functioning because of altered proportions of the available functional traits of species (Schmera et al., 2019). Consequently, the ability of river ecosystems to provide ecosystem services to human populations is decreased, including losses in climate regulation, water quality, carbon regulation, water and food provisioning, recreation, and disease prevention.

The correlations between the biological condition of rivers and the indicators of anthropogenic alterations showed that measures aimed at improving terrestrial and aquatic ecosystems (such as increased natural forest, where it existed, and freshwater protected areas) can be effective in improving the biological condition of rivers (see McGarvey et al., 2021). Indeed, up to now inland waters remain poorly represented in protected areas namely in Europe (EEA, 2020) and Australia and previous studies showed that the protection measures strictly focused on terrestrial systems confer little benefit to freshwater species (Chessman, 2013; Leal et al., 2020). In other regions of the world, freshwater protected areas do exist, such as the National Freshwater Priority Areas in South Africa (NFEPA, Nel et al., 2011) and existing studies indicate that they had a positive effect on the ecological status of rivers (Nel et al., 2007). This is also aligned with recent calls for joint efforts across different realms, such as terrestrial, freshwater, and estuarine/marine ecosystems (Arthington, 2021).

Thus, the establishment of a wider network of protected areas for rivers should be incentivized globally with actions focused on the preservation of aquatic and associated‐terrestrial biodiversity, riparian forests, and ecosystem processes. It is especially important to consider processes that involve transfers of matter, energy, water and organisms between land and water and along the river longitudinal gradient (Feio, Serra, et al., 2021). This is aligned with the priority actions in the Emergency Recovery Plan by Tickner et al. (2020), which recommend the acceleration of the implementation of environmental flows, restoration of freshwater connectivity and critical habitats, improvement of water quality and fisheries management, and prevention of species invasions.

To guarantee the success of any of these approaches it is also essential to promote and incentivize the values of nature and biodiversity across all sectors of society using integrative approaches drawing on diverse disciplines and integrating values and ethics (Feio, Ranta, et al., 2021; Odume & de Wet, 2019). Human societies are growing more disconnected from nature, however, environmental education, citizen science and citizen involvement in natural areas governance can increase ecosystem knowledge and improve the understanding of the services a healthy ecosystem provides humanity, reduce fears of nature, increase nature preservation, and improve social cohesion (Aslanimehr et al., 2018; Feio et al., 2022; Mattiijssen et al., 2018). In addition, including nature and biodiversity integrity as positive indicators in the HDI could contribute to awareness‐raising among decision‐makers and users of catchments and river ecosystem services.

Global partnerships, like the one of this study, which use existing data to conduct global assessment and comparisons can be used to support national action in understudied areas (see also Eriksen et al., 2021; Maasri et al., 2021; van Rees et al., 2021). The establishment and implementation of legal national and international frameworks, like the European Water Framework (EC, 2000) or the US‐EPA's National Rivers and Streams Assessment (Paulsen et al., 2020) is also a strong incentive for the development of common standards and official nation‐wide monitoring programs.

In summary, our large‐scale analysis, that incorporated the greatest amount of data from the Global South so far, showed that more than half of the rivers/water bodies studied globally were below Good biological quality. A better biological quality of rivers was associated to better water quality, increased forested areas, and higher percentages of protected areas. The degradation of rivers was not related to the history of non–First Nations modifications/human occupation and arid and equatorial regions are the ones more affected by poorer biological quality. Finally, the degradation of rivers affects more strongly fish assemblages than the macroinvertebrates.

## AUTHOR CONTRIBUTIONS

Maria João Feio and Robert M. Hughes conceived the research and completed the first and revised drafts and Maria João Feio and Sónia R. Q. Serra performed data analyses. All authors acquired and interpreted data, substantively revised the drafts, or both, and have approved the submitted version. We agree to be personally accountable for our own contributions and to ensure that questions related to the accuracy or integrity of any part of this work, even ones in which we were not personally involved, are appropriately investigated, resolved, and documented in the literature.

## CONFLICT OF INTEREST

All authors declare that they don't have any conflicts of interest.

## Supporting information


Data S1

## Data Availability

The data that supports the findings of this study are available in Dryad at https://doi.org/10.5061/dryad.pnvx0k6r9
