# Supplementary material for: Fish and macroinvertebrate assemblages reveal extensive degradation of the world's rivers
Source: Glob Chang Biol. 2022 Oct 17;29(2):355–74. doi: 10.1111/gcb.16439 (PMC10091732; doi:10.1111/gcb.16439)
Supplement: Supplementary file 1 — Data S1 [file GCB-29-355-s001.docx]

**Table S1.** Data sources.

| **Nation** | **Data base or other source** |
| --- | --- |
| South Africa | Department of Waters Affairs & Forestry (DWAF) 2007. Rivers Database (unpublished) |
| Nigeria | Keke, U.N. et al. (2021): <https://doi.org/10.1016/j.ecolind.2021.107868>  Tonkin, J.D. et al. (2016): <https://doi.org/10.1007/s10531-016-1101-2> |
| South Korea | Water Environment Information System (<http://water.nier.go.kr/web/bioMeasure?pMENU_NO=1>), Ministry of Environment |
| China | Ding, N. et al. (2017) <https://doi.org/10.1016/j.scitotenv.2016.09.053>  Wei, H. et al. (2020) <https://doi.org/10.17520/biods.2019359>  Chen, K. et al. (2019). <https://doi.org/10.1007/s10750-019-3970-3>  Chen et al. (2014) <https://doi.org/10.1016/j.ecolind.2013.07.006>  Chen K. et al. (Unpublished data) |
| Japan | National Census on River Environments <http://www.nilim.go.jp/lab/fbg/ksnkankyo/>;  Sueyoshi, M. et al. (2016) <https://doi.org/10.18960/hozen.21.2_167> |
| Lower Mekong | Data collected by Mekong River Commissions:  [www.mrcmekong.org](http://www.mrcmekong.org) |
| Nepal | Tachamo Shah, R.D et al. (2020) <https://doi.org/10.1016/j.ecolind.2019.105735>  Tachamo Shah, R.D et al. (2020) <https://doi.org/10.3390/geosciences10040150>  Tachamo Shah, R.D et al. (2019) <https://doi.org/10.25919/5e73b328f1c5f>  Tachamo Shah, R.D. et al. (2015) https:// doi.org/10.1007/s10584-015-1417-z  Tachamo Shah, R.D., Shah D.N. (2012) <https://doi.org/10.1016/j.ecolind.2012.04.001>  Tachamo Shah, R.D. 2011. ISBN-10 : ‎9783845400853  Shah, D.N. 2011. ISBN 978-38443-1-366-6  Unpublished data (Tachamo Shah, R.D. & Shah, D.N.) |
| European Union, UK and Norway | WISE database (European Environment Agency):  <https://www.eea.europa.eu/data-and-maps/data/wise-wfd-4> |
| USA | NARS (USA National Aquatic Resource Surveys):  <https://www.epa.gov/national-aquatic-resource-surveys/data-national-aquatic-resource-surveys> |
| Mexico | \| Torres-Olvera, M. et al. 2018. <https://doi.org/10.3856/vol46-issue5-fulltext-8> \| \| --- \| \| Pérez-Munguia, R.M. et al. (2019). ISSN- 2448-475X <https://www.socmexent.org/entomologia/revista/2019/EC/EC%20414-420.pdf> \| \| Piñon Flores, M.A. et al. (2014). ISSN-0034-7744 <https://www.scielo.sa.cr/scielo.php?script=sci_arttext&pid=S0034-77442014000600015> \| \| Ramirez Herrejon, J.P. et al. (2012) ISSN-0034-7744 <https://tropicalstudies.org/rbt/attachments/volumes/vol60-4/24_Ramirez_Indices_biologicos.pdf> \| \| Moncayo-Estrada, R. et al. (2014) <https://doi.org/10.1002/rra.2774> \| \| Cabrera Borja, Y. (2016) Thesis. Universidad Michoacana de San Nicolás de Hidalgo \| \| Mercado-Silva, N. et al. (2001) Thesis. University of Wisconsin, Madison. \| \| Mercado-Silva, N. (unpublished database) \| |
| Canada | Environment and Climate Change Canada (2021) Canadian Aquatic Biomonitoring Network (CABIN) Database. Retrieved on 21^st^ May 2021, from  <https://www.canada.ca/en/environment-climate-change/services/canadian-aquatic-biomonitoring-network/database.html> |
| New Zealand | <https://www.lawa.org.nz/> |
| Australia | Data gathered by the Department of Primary Industries, Parks, Water and Environment, Tasmania; Environmental Monitoring Unit, EPA Victoria; NSW Department of Planning, Industry and Environment; Water Science Branch, Department of Water WA (https://ausrivas.ewater.org.au/index.php/contact)  Fish and macroinvertebrate data from the Murray-Darling Basin: <https://data.gov.au/dataset/ds-dga-7826d7c9-bcc5-48c0-832a-66aaedfe7b0f/details?q=> |
| Brazil | Alves, C.B.M. & Pompeu, P.S. (2015-2017 campaigns - unpublished database)  Carvalho, D.R. et al. (2017) [10.1016/j.ecolind.2017.02.032](https://doi.org/10.1016/j.ecolind.2017.02.032)  Martins, R.T. et al. (2021) doi.org/10.1016/j.ecolind.2021.107773  Feio, M.J. et al. (2015) [http://dx.doi.org/10.1002/rra.2716](https://signal2domain.online/click?redirect=http%3A%2F%2Fdx.doi.org%2F10.1002%2Frra.2716&dID=1633724105910&linkName=http://dx.doi.org/10.1002/rra.2716)  Silva, D.R.O. et al. 2017) [http://dx.doi.org/10.1016/j.ecolind.2017.06.017](https://signal2domain.online/click?redirect=http%3A%2F%2Fdx.doi.org%2F10.1016%2Fj.ecolind.2017.06.017&dID=1633724105910&linkName=http://dx.doi.org/10.1016/j.ecolind.2017.06.017)  Martins, I. et al (2020) [https://doi.org/10.1016/j.ecolind.2019.105953](https://signal2domain.online/click?redirect=https%3A%2F%2Fdoi.org%2F10.1016%2Fj.ecolind.2019.105953&dID=1633724105910&linkName=https://doi.org/10.1016/j.ecolind.2019.105953)  Callisto, M. & Macedo D. (unpublished macroinvertebrate data, 2019) |
| Bolivia | Data from Moya, N. et al. (2011).  [doi.org/10.1016/j.ecolind.2010.10.012](https://doi.org/10.1016/j.ecolind.2010.10.012) |

**Table S2.** Methods for sampling benthic macroinvertebrates and fish and assessing the biological quality of riverine sites in the study areas. RCA: method based on the Reference Condition Approach (i.e., boundaries adjusted to the reference conditions for that region/river type/environmental characteristics). Sampling approach: hand net (kick and sweep sampling). Index type: multimetric index (MMI), predictive taxa richness model (PM), biotic index (BI, based on organic pollution tolerance), other (taxa diversity index or EPT genus richness). NA – information is not available.

| **Study region** | Macroinvertebrates | | | Fish | | |
| --- | --- | --- | --- | --- | --- | --- |
|  | **RCA** | **Sampling** | **Index** | **RCA** | **Sampling** | **Index** |
| South Africa | yes | hand net | BI | - | - | - |
| Nigeria | yes | hand net | BI | - | - | - |
| South Korea | yes | Surber | BI | yes | cast nets, hand net | MMI |
| China | yes | hand net or Surber | PM, MMI | - | - | - |
| Japan | yes | Surber | Other | yes | electrofishing, cast-nets, seines, hand nets, and gill nets. | Species richness |
| Lower Mekong - Laos, Thailand, Vietnam, Cambodia | yes | hand net | BI | - | - | - |
| Nepal | yes | hand net | BI | - | - | - |
| Austria | yes | hand net | MMI, BI | yes | electrofishing | MMI |
| Belgium | yes | hand net | MMI, BI | yes | electrofishing | MMI |
| Bulgaria | yes | hand net | BI | yes | electrofishing | MMI |
| Croatia | yes | several devices | BI | - | - | - |
| Cyprus | yes | hand net | MMI | - | - | - |
| Czechia | yes | hand net | BI | yes | electrofishing | MMI |
| Denmark | yes | hand net | BI | yes | electrofishing | MMI |
| Estonia | yes | hand net | BI | yes | electrofishing | MMI |
| Finland | yes | hand net | BI | yes | electrofishing | MMI |
| France | yes | hand net | BI | yes | electrofishing | Predictive MMI |
| Germany | yes | hand net, Surber or Hess sampler | MMI | yes | electrofishing | MMI |
| Greece | yes | several devices | MMI | yes | electrofishing | Predictive MMI |
| Hungary | yes | hand net | BI | yes | electrofishing | MMI |
| Ireland | yes | hand net | BI | yes | electrofishing | Predictive MMI |
| Italy | yes | hand net | MMI | yes | electrofishing | MMI |
| Lithuania | yes | hand net | MMI | yes | electrofishing | MMI |
| Luxembourg | yes | Surber or Hess sampler | BI | yes | electrofishing | Predictive MMI |
| Latvia | yes | hand net | BI | yes | electrofishing | Predictive MMI |
| Netherlands | yes | hand net | MMI | yes | electrofishing | MMI |
| Norway | yes | hand net | BI | yes | NA | NA |
| Poland | yes | hand net | MMI, BI | yes | electrofishing | Predictive MMI |
| Portugal | yes | hand net | MMI | yes | electrofishing | MMI |
| Romania | yes | Ponar grab, Surber sampler, Hand net | BI | yes | electrofishing | Predictive MMI |
| Slovakia | yes | hand net | MMI | yes | electrofishing | MMI |
| Slovenia | yes | hand net, Surber or Hess sampler | MMI | yes | electrofishing | MMI |
| Spain | yes | hand net | MMI, BI | yes | electrofishing | MMI |
| Sweden | yes | hand net | MMI | yes | electrofishing | Predictive MMI |
| United Kingdom | yes | hand net | PM | yes | electrofishing | PredictiveMMI |
| USA | yes | hand net | MMI | yes | electrofishing | Regional  MMIs |
| Mexico | yes | hand net | MMI | yes | electrofishing, gillnets and/or seines | MMI |
| Canada | yes | hand net | PM | - | - | - |
| New Zealand | yes | hand net | BI | - | - | - |
| Murray Darling basin – Australia | yes | hand net | PM | yes | electrofishing | PM |
| Australia (SE, SW coasts, Tasmania) | yes | hand net | PM | - | - | - |
| Brazil-Cerrado | yes | hand net | MMI | yes | hand net, seine | MMI |
| Brazil-Amazon | yes | hand net | other | yes | hand net, seine | other |
| Bolivia | yes | Surber | Predictive MMI | - | - | - |

**Table S3.** Pearson correlations (r, p and df) and Lin’s Concordance Correlation (Lin’s R) among biological quality classifications (% of sites in Good, Moderately Impaired or Severely Impaired condition) of the study areas, and the Pearson’s correlations between the biological quality classifications and the global indices: Human Development (HDI), Human Footprint (HF), and the Sustainable Development Goals % rivers with good ambient water quality – Good water quality; % protected freshwater key biodiversity areas – Protected freshwater areas; and % of forest area annual net change rate – Forest change). Inv. = benthic macroinvertebrates; Mod. = Moderate; Sev. = Severely

| **Lin’s concordance** | ***Lin’s R*** | ***Lbound*** | | ***Ubound*** | |
| --- | --- | --- | --- | --- | --- |
| Good Inv. - Good Fish | 0.58 | 0.32 | | 0.76 | |
| Mod. Impaired Inv. – Mod. Impaired Fish | 0.46 | 0.13 | | 0.70 | |
| Sev. Impaired Inv. – Sev. Impaired Fish | 0.56 | 0.20 | | 0.79 | |
| **Pearson correlations** (pairs of variables) | ***r*** | | ***p*-value** | | ***df*** |
| Good Inv. - Good Fish | 0.62 | | <0.001 | | 40 |
| Mod. Impaired Inv. – Mod. Impaired Fish | 0.47 | | 0.002 | | 40 |
| Sev. Impaired Inv. – Sev. Impaired Fish | 0.63 | | <0.001 | | 40 |
| Good Inv. - HF | -0.09 | | 0.497 | | 62 |
| Good Fish - HF | 0.14 | | 0.376 | | 40 |
| Mod. Impaired Inv. - HF | 0.05 | | 0.714 | | 62 |
| Mod. Impaired Fish - HF | -0.42 | | 0.006 | | 40 |
| Sev. Impaired Inv. - HF | 0.05 | | 0.706 | | 62 |
| Sev. Impaired Fish - HF | 0.14 | | 0.373 | | 40 |
| Good Inv. - HDI | 0.11 | | 0.398 | | 62 |
| Good Fish - HDI | -0.17 | | 0.276 | | 40 |
| Mod. Impaired Inv. - HDI | -0.09 | | 0.485 | | 62 |
| Mod. Impaired Fish - HDI | -0.10 | | 0.535 | | 40 |
| Sev. Impaired Inv. - HDI | -0.06 | | 0.658 | | 62 |
| Sev. Impaired Fish - HDI | 0.37 | | 0.015 | | 40 |
| Good Inv. - Good water quality | 0.43 | | 0.003 | | 45 |
| Good Fish - Good water quality | 0.30 | | 0.084 | | 33 |
| Mod. Impaired Inv. - Good water quality | 0.02 | | 0.892 | | 45 |
| Mod. Impaired Fish - Good water quality | 0.04 | | 0.802 | | 33 |
| Sev. Impaired Inv. - Good water quality | -0.52 | | <0.001 | | 45 |
| Sev. Impaired Fish - Good water quality | -0.36 | | 0.032 | | 33 |
| Good Inv. - Forest change | 0.17 | | 0.189 | | 62 |
| Good Fish - Forest change | 0.30 | | 0.058 | | 40 |
| Mod. Impaired Inv. - Forest change | -0.26 | | 0.034 | | 62 |
| Mod. Impaired Fish - Forest change | -0.28 | | 0.074 | | 40 |
| Sev. Impaired Inv. - Forest change | -0.01 | | 0.947 | | 62 |
| Sev. Impaired Fish - Forest change | -0.18 | | 0.265 | | 40 |
| Good Inv. - Protected freshwater areas | 0.14 | | 0.253 | | 62 |
| Good Fish - Protected freshwater areas | 0.21 | | 0.189 | | 40 |
| Mod. Impaired Inv. - Protected freshwater areas | 0.16 | | 0.203 | | 62 |
| Mod. Impaired Fish - Protected freshwater areas | -0.14 | | 0.377 | | 40 |
| Sev. Impaired Inv. - Protected freshwater areas | -0.31 | | 0.011 | | 62 |
| Sev. Impaired Fish - Protected freshwater areas | -0.16 | | 0.304 | | 40 |


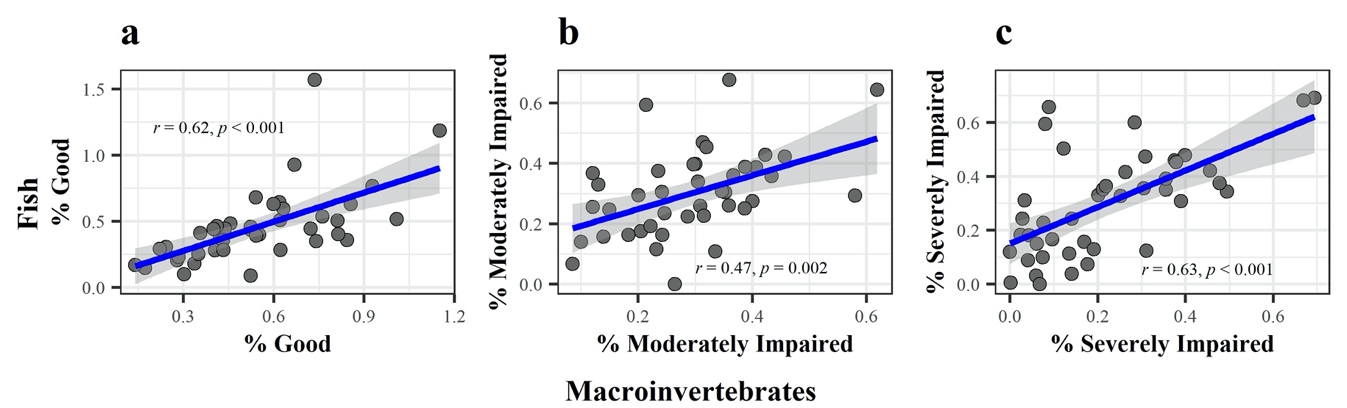


Lin’s R=0.58

Lin’s R=0.46

Lin’s R=0.56

**Fig. S1** Percent of sites in Good, Moderately Impaired, and Severely Impaired condition based on macroinvertebrates versus fish assemblages. Data were arcsine transformed. The Pearson correlation coefficient (*r*) is presented and a two-tailed t-test was used to determine the significance level (*p*<0.05 - blue line). The grey area defines the 95% confidence level interval for predictions from a linear model. In addition, Lin’s estimate of concordance correlation (Lin’s R) is presented above each comparison.


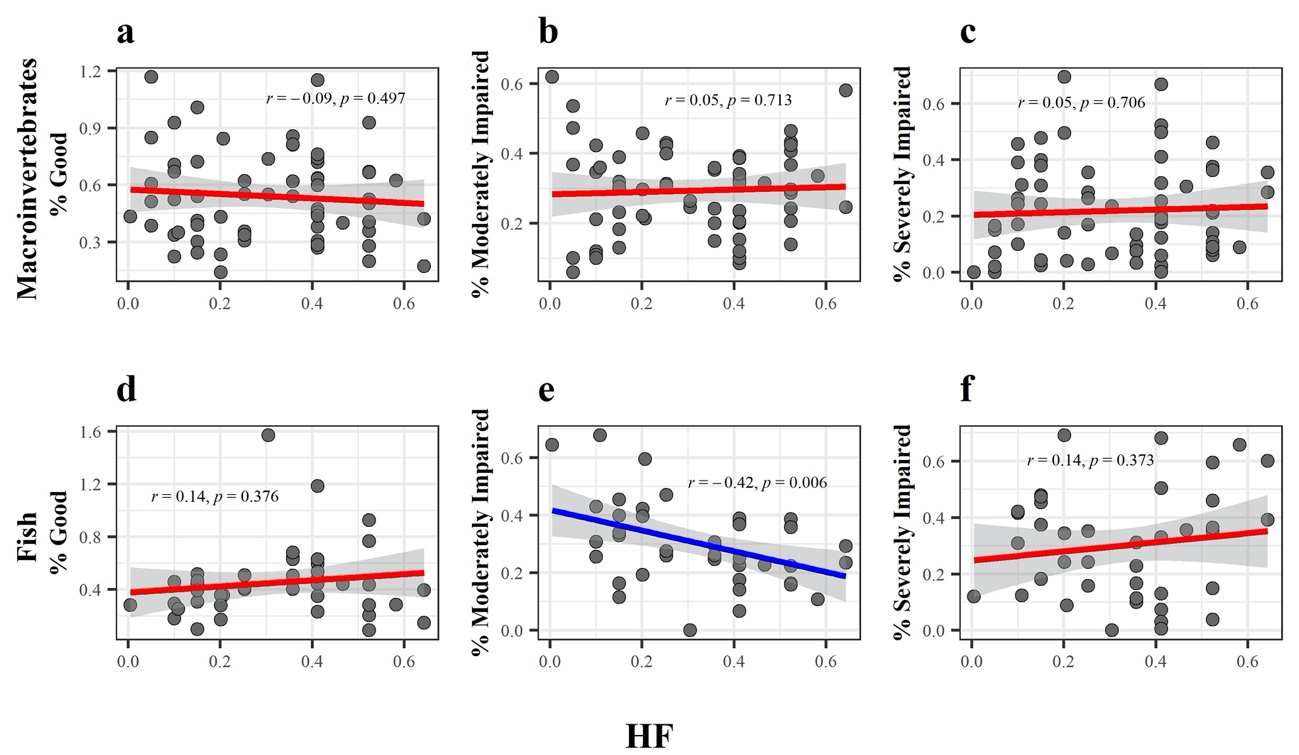


**Fig. S2** Percent of sites in Good, Moderately Impaired, and Severely Impaired condition based on macroinvertebrates (above) and fish assemblages (below) versus regional Human Footprint (HF) index scores. Data were arcsine transformed. The Pearson correlation coefficient (*r*) is presented and a two-tailed t-test was used to determine the significance level (*p*<0.05 - blue line). The grey area defines the 95% confidence level interval for predictions from a linear model.

**
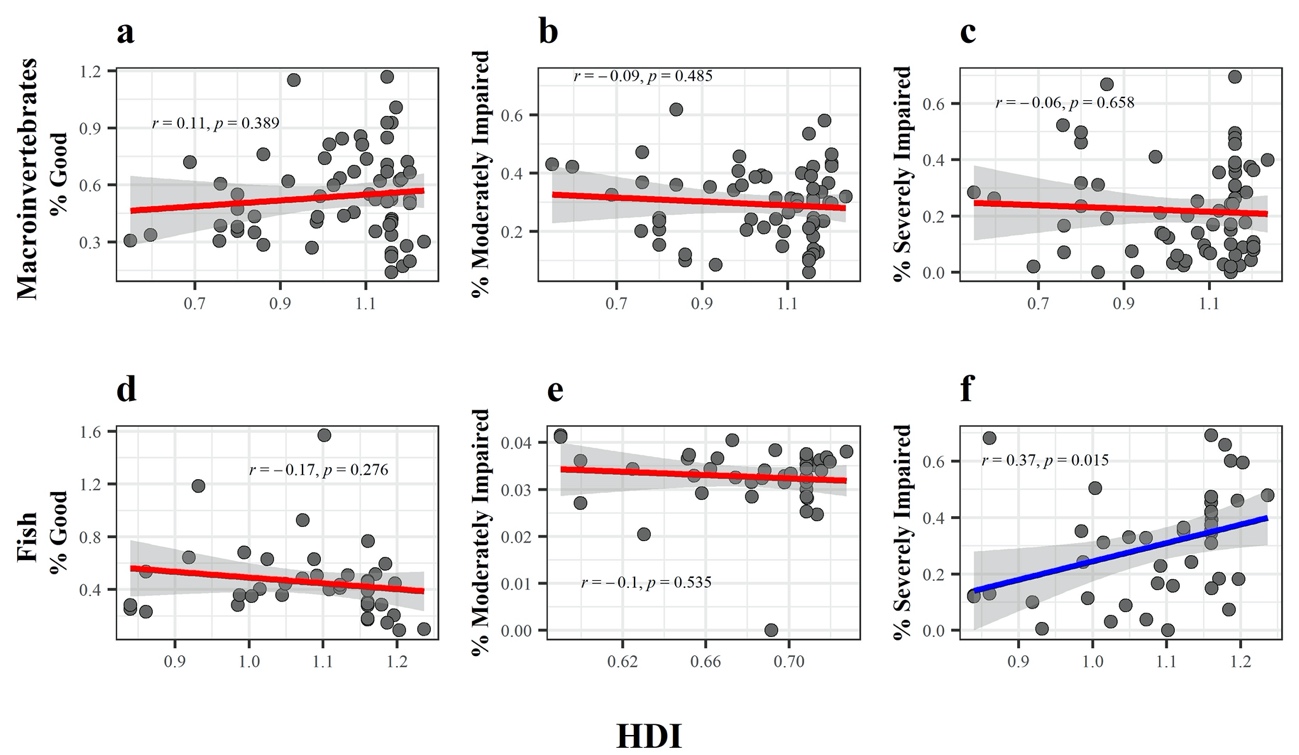
**

**Fig. S3** Percent of sites in Good, Moderately Impaired, and Severely Impaired condition based on macroinvertebrates (above) and fish assemblages (below) versus regional Human Development Index (HDI) scores. Data were arcsine transformed. The Pearson correlation coefficient (*r*) is presented and a two-tailed t-test was used to determine the significance level (*p*<0.05 - blue line). The grey area defines the 95% confidence level interval for predictions from a linear model.


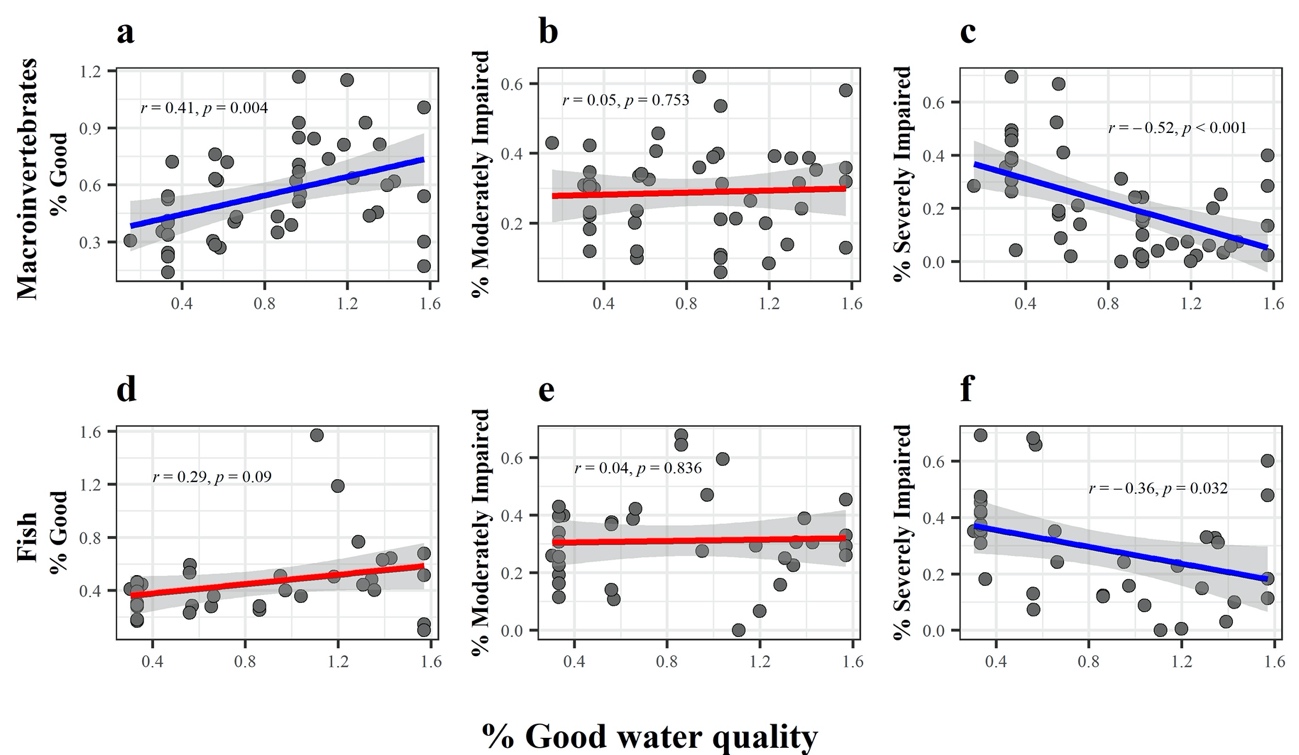


**Fig. S4** Percent of sites in Good, Moderately Impaired, and Severely Impaired condition based on macroinvertebrates (above) and fish assemblages (below) versus regions with Good ambient water quality. Data were arcsine transformed. The Pearson correlation coefficient (r) is presented and a two-tailed t-test was used to determine the significance level (*p*<0.05 - blue line). The grey area defines the 95% confidence level interval for predictions from a linear model.


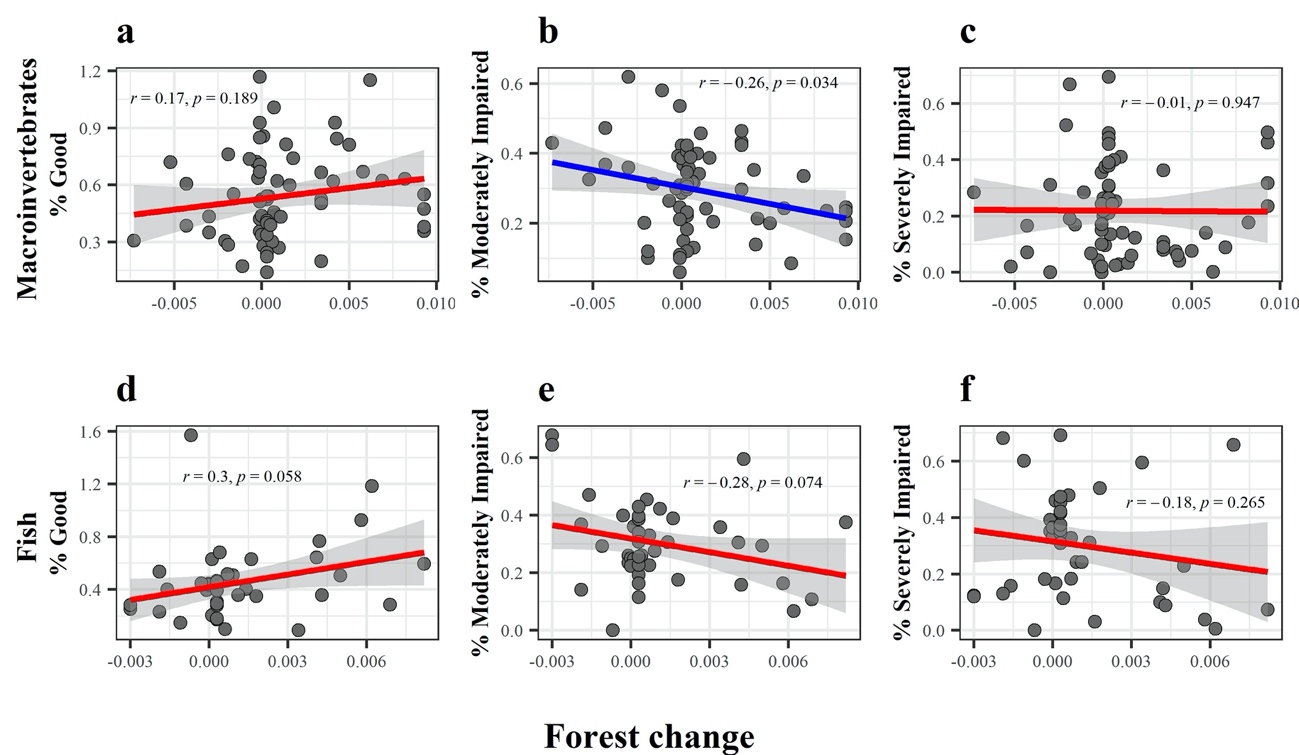


**Fig. S5** Percent of sites in Good, Moderately Impaired, and Severely Impaired condition based on macroinvertebrates (above) and fish assemblages (below) versus the % of net change in forest area (Forest change). Data were arcsine transformed. The Pearson correlation coefficient (*r*) is presented and a two-tailed t-test was used to determine the significance level (*p*<0.05 - blue line). The grey area defines the 95% confidence level interval for predictions from a linear model.

**
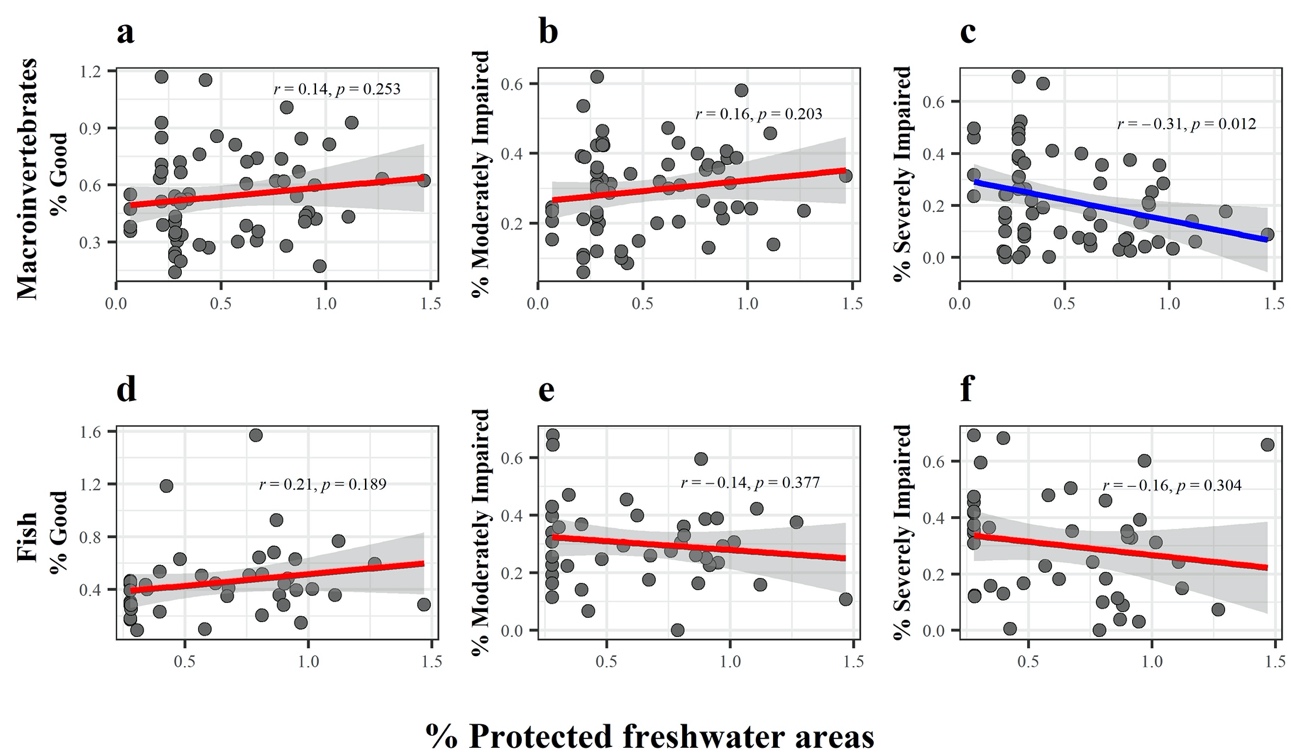
Fig. S6** Percent of sites in Good, Moderately Impaired, and Severely Impaired condition based on macroinvertebrates (above) and fish assemblages (below) versus the % of protected freshwater area. Data were arcsine transformed. The Pearson correlation coefficient (*r*) is presented and a two-tailed t-test was used to determine the significance level (*p*<0.05 - blue line). The grey area defines the 95% confidence level interval for predictions from a linear model.
